# *LevelWAN*: a cost-effective, open-source IoT system for water level monitoring in highly dynamic aquatic environments

**DOI:** 10.1016/j.ohx.2025.e00685

**Published:** 2025-08-07

**Authors:** Ilane Cherif, Frédéric Cherqui, Franck Perret, Bastien Bourjaillat, Lionel Lord, Jean-Luc Bertrand-Krajewski, Nicolas Walcker, Maria Gisi, Laëtitia Bacot, Oldrich Navratil

**Affiliations:** a Université de Lyon, INSA Lyon, DEEP EA 7429, 11 rue de la Physique, F-69621 Villeurbanne cedex, France; b Université de Lyon, UMR 5600 CNRS-Environnement Ville Société, Université Lumière Lyon 2, 5 avenue Pierre Mendès-France, Bron Cedex F-69635, France; c Univ Lyon, INSA Lyon, DEEP, EA7429, 69621 Villeurbanne, France; d School of Agriculture, Food and Ecosystem Sciences, The University of Melbourne, Burnley, VIC 3121, Australia; e GRAIE, 66 Boulevard Niels Bohr, CS 52132, 69100 Villeurbanne cedex, France

**Keywords:** Real-time monitoring, Arduino, Field measurements, Hydrology, Environmental education, River

## Abstract

The deployment of low-cost network sensors (LCNS) for environmental monitoring has become increasingly prevalent in recent years, offering a cost-effective solution for enhancing spatial sensor coverage while minimizing financial constraints. This study presents *LevelWAN*, a water level monitoring system specifically designed for highly dynamic aquatic environments such as rivers, ponds or lakes. *LevelWAN* is an open-source, robust, and cost-effective Internet of Things (IoT)-based monitoring solution incorporating an ultrasonic sensor. The electronic components were carefully selected for their affordability, reliability, and performance. The system underwent a fully autonomous, long-term (3-year) field test in a challenging and highly dynamic environment – a sewer system – to validate its robustness. Its accuracy was assessed against a high-precision professional device, demonstrating an error margin of less than 1 cm. Additionally, *LevelWAN* was developed with a user-friendly design to facilitate accessibility for non-experts, aligning with the needs of citizen science initiatives and educational applications.

## Graphical abstract

**Figure:**
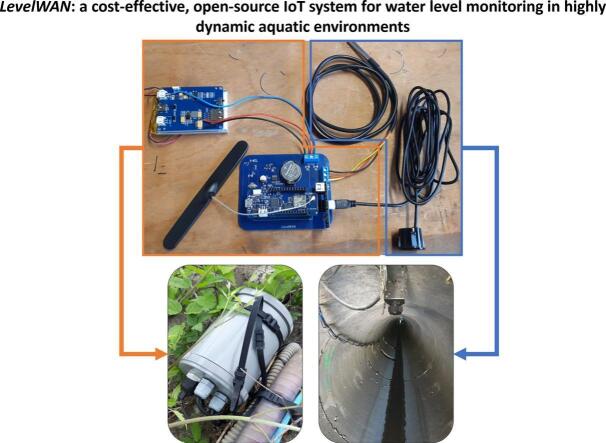

## 1 Hardware in context

Water level is a fundamental parameter in hydraulic and environmental studies. Although simple, it is of strategic importance as it provides critical insight into the spatial and temporal dynamics of various water bodies, including rivers, lakes, ponds, groundwater systems, artificial stormwater retention or infiltration basins, wastewater systems and water storage facilities (ranging from dams to irrigation reservoirs). Accurate measurement of water levels is essential for understanding aquatic environments, managing water quality and quantifying water resources, such as river discharge or groundwater level fluctuations.

The operational and scientific issues involved in measuring water levels also have economic and political dimensions, especially when it comes to quantifying and allocating water resources shared by different stakeholders or several countries (*e.g.* [1]). This strategic monitoring of the environment has left its mark on the history of environmental science. For example, as early as 3000 BCE, measurements of the level of the Nile taken at the Nilometric Station in Alexandria were used to calculate agricultural taxes based on the intensity of annual floods. These floods were essential to provide the silt and water needed for bountiful harvests [2]. Much later, in 1831, Henry Palmer revolutionized these practices by developing the first continuous recorder of river levels, combining a cylindrical roller and a float. Thanks to their simplicity, reliability and strategic importance, these measuring systems were widely adopted and used until the middle of the twentieth century. However, instrumentation requires extensive and costly infrastructure as well as regular and expensive maintenance. Since the 1980s and 1990s, they have mainly been replaced by piezometric, ultrasonic or radar sensors [3]. These systems have the advantage of being easy to install in the field, more robust, more accurate (often to within one centimeter) and require less maintenance.

The principle of ultrasonic water level measurement is based on measuring the transit time of an acoustic wave emitted by the sensor above the free surface of the water and reflected by the air–water interface towards the sensor receiver [4]. Knowing the speed of sound in air, the travel time can be converted into a distance between the sensor and the air–water interface, which can then be used to calculate the water level if the depth of the river, canal or reservoir is known. This measurement is non-intrusive, which is of great interest for monitoring water levels in rivers, especially during floods, as well as in urban water systems. However, the large-scale use of these traditional ultrasonic sensors in highly dynamic water systems remains limited due to their cost (>2000 euros for a sensor with a data logger without data transmission). The only major obstacle to the use of so-called traditional dataloggers and sensors in environmental science is their very high cost, which prevents the spatial distribution of measurement points. Covering a study area more densely would enable us to better understand natural processes at most “hot spots” during most “hot moments” [5]. The price solution adopted by many researchers is the use of low-cost educational electronics, which, according to the current literature, is becoming increasingly common in research, but still has its limitations.

Today, low-cost sensors can measure water levels (and other liquids) for less than 200 Euros (= 211.7 USD), a tenth of the price of a traditional sensor. Many turnkey solutions are currently available on the market (e.g. Nivus GmbH i-series), providing pre-built and fully functional systems. These solutions are practical, as they enable rapid deployment without the need for further development or configuration. However, they present certain limitations, particularly in terms of customization for research applications. Specifically, they are neither modular nor open-source, which restricts the ability of users to make tailored adjustments or adapt the system to specific requirements. This lack of flexibility can hinder the system’s capacity for evolution and adaptation in dynamic and ever-changing environments. On the other hand, there are systems based on inexpensive open-source microcontrollers [[6], [7], [8], [9], [10]], such as Arduino [11], Raspberry Pi [7] or Adafruit [8], combined with other low-cost methods such as 3D printing to significantly reduce the cost compared to using proprietary tools for research [11].

To adapt these low-cost sensors for autonomous field experiments, they need to be connected either to an SD card to store data only locally [7,8], or to a telecommunications module with an antenna to send data online [6], or both. The choice between these two methods depends on the objectives, context and location of the site: local recording is not suitable for real-time remote monitoring, and online methods depend heavily on the strength of the signal sent by the device (linked to the distance from the nearest gateway). Fortunately, today's low-cost educational microcontrollers often have online connectivity and compatibility with low-cost SD card modules (e.g., Adafruit [8], Arduino MKR WAN 1300/1310 [12]). In addition to simply reducing purchase costs, low-cost water level metrology makes it possible to: i) distributed monitoring, e.g., a large-scale approach by installing these sensors at numerous points in a river or groundwater network, ii) calibration and testing of models (hydrological, hydraulic) of water tables, rivers and rainwater management techniques, iii) live monitoring of the measurement systems themselves (greater robustness), iv) regaining control over the measurement itself and moving in the direction of open and frugal science [13], and v) the possibility of opening up these approaches to citizen science [14,15]. With the development of the IoT, users can visualize their measurements in near-real time, which is rewarding and involves users even more in open science and participatory science projects [16].

This article describes *LevelWAN,* a new low-cost, open-source IoT water level measurement system based on an ultrasonic sensor, and adapted to harsh and highly dynamic aquatic environment. *LevelWAN* was developed to become a base for low-cost sensor users to build upon, especially regarding the datalogging part which provides a new way to make high frequency measurements with a very low power consumption. It follows the philosophy of frugal science [13] by repurposing an industrial sensor — in this case, a car sensor — for environmental and citizen science applications. This makes it an excellent practical example for environmental education, particularly in schools. We present the different steps to build and program this system, and several field tests led at various experimental sites over several months (e.g., robustness of the system, quality of the LoRaWAN connection, timestamp precision, and power consumption).

## 2 Hardware description

### 2.1 System overview

**Fig. 1:**
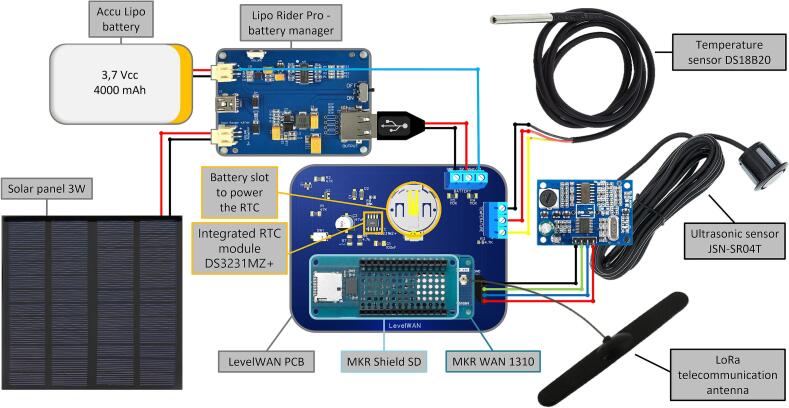
Wiring diagram of the LevelWAN system with sensors, microcontroller, power supply and PCB.

**Fig. 2:**
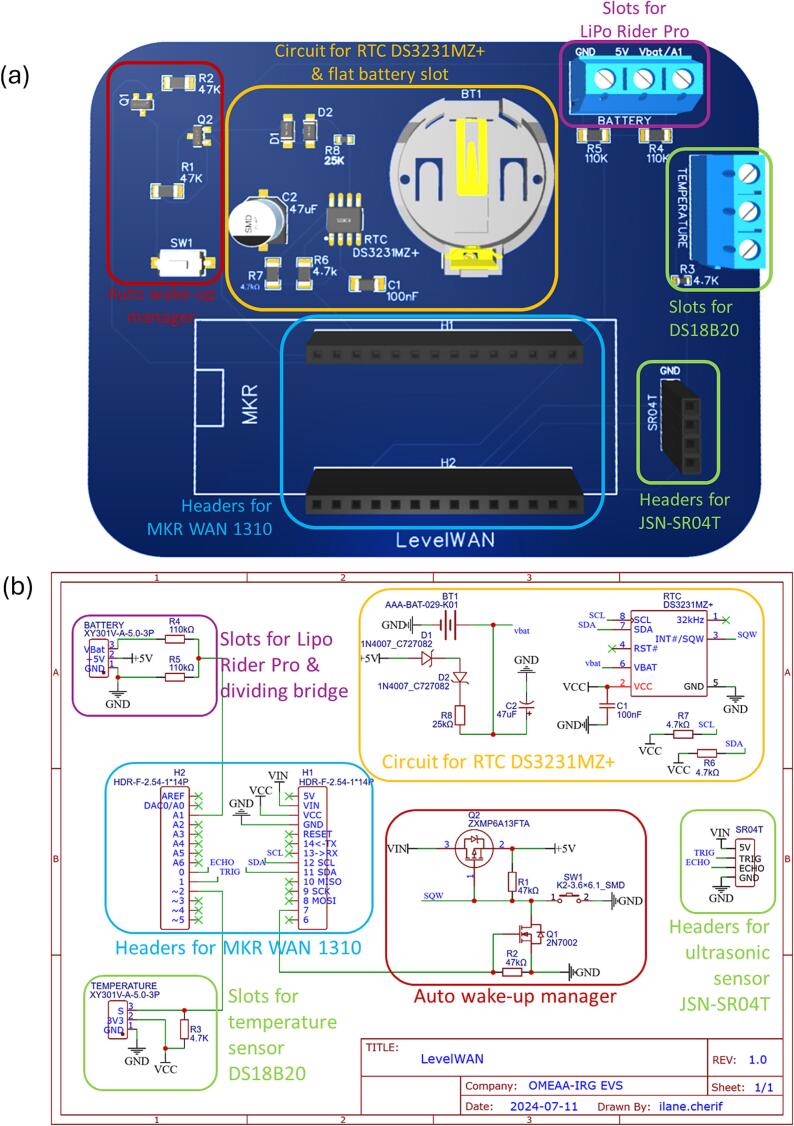
LevelWAN PCB (a) and its schematic (b) with connector pins for i) the ultrasonic sensor JSN-SR04T (in green), the temperature sensor DS18B20 (in green), the battery manager (in purple), the MKR WAN 1310 (in blue) and the RTC DS3231 (in yellow). The wake-up manager is part of the PCB (in red). (For interpretation of the references to colour in this figure legend, the reader is referred to the web version of this article.)

*LevelWAN* is based on an ultrasonic sensor. It includes six modules (Fig. 1, Fig. 2):

• A waterproof ultrasonic distance sensor JSN-SR04T, with a measurement range from 0.2 m to 1.9 m, and a 95 % expanded uncertainty of ± 7 mm empirically estimated during laboratory tests conducted by Cherqui *et al.* [17]. The distance calculation was based on the time-of-flight measured by a JSN-SR04T sensor and the temperature-corrected sound velocity using a temperature sensor with a ± 0.5 °C uncertainty announced by the manufacturer;
• A waterproof temperature sensor DS18B20, with a measurement range from −10 °C to 85 °C, and a 95 % expanded uncertainty of ± 0.5 °C [18], similar to the sensor used by Cherqui *et al.* [17];
• A datalogger consisting of a microcontroller MKR WAN 1310 with a LoRa telecommunication antenna, a MKR MEM SHIELD SD (with an SD card reader), and a 16 GB SD card;
• A power supply with a small solar panel SOL3W (3Wc, 5.5 V/540 mA, size: 160 × 138 mm), a LiPo Rider Pro solar panel regulator (SEEED Studio), and a LiPo battery (3.7 V, 4000 mAh/unit, SEEED Technology Corp.);
• A Printed Circuit Board (PCB) with an integrated auto wake-up module, designed for this project (full schematic in Fig. 2 and design file in Table 1); It prevents power consumption between two data acquisitions by completely shutting down the system rather than allowing the microcontroller to enter sleep mode. The current consumption was estimated to be 47 µA, compared to 104 µA with the pre-programmed (but not enabled by default) “VERY_LOW_POWER” mode of the MKR WAN 1310 [11];

**Table 1:** Design files.

**Design file name**	**File type**	**Open source license**	**Location of the file**
Design file 1	PCB reproduction files (.zip file)	GNU General Public License (GPL) 3.0	In the OSF repository
Design file 2	Arduino script (.ino file)	GNU General Public License (GPL) 3.0	In the OSF repository
Design file 3	Video tutorial (.mov file)	GNU General Public License (GPL) 3.0	In the OSF repository
Design file 4	Google sheet script (.txt/JavaScript file)	GNU General Public License (GPL) 3.0	In the OSF repository
• A Real-Time Clock (RTC) DS3231 to set alarms [19] for maintaining precise monitoring intervals and timestamps while conserving battery power between measurements.

**Fig. 3:**
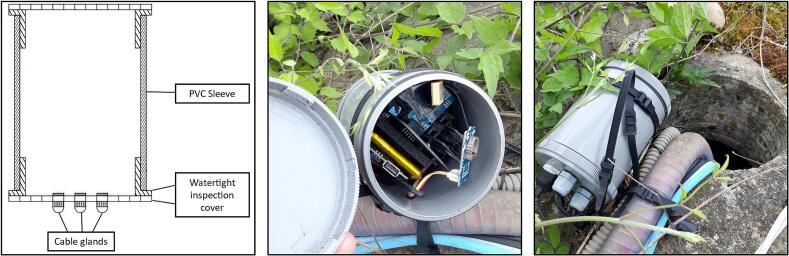
Encapsulation of the LevelWAN electronic elements inside a low-cost waterproof PVC case and its field installation.

The electronic components are housed inside a homemade PVC waterproof case to protect them from environmental hazards (Fig. 3). The robustness of our casing system was demonstrated by three years of continuous field measurements without any device deterioration or moisture issues. The monitoring system is designed for near real-time monitoring, with data communicated to The Things Network (TTN) via LoRaWAN. This communication protocol, and therefore the microcontroller, were chosen because of the low power of the LoRaWAN signal (1 to 10 mW) and its long detection range (ideally up to ∼ 15 km). The data is stored in a Google Spreadsheet and time series (water level, temperature, battery level) are displayed on an online platform (https://opendataeau.org). *LevelWAN* is easy to assemble and suitable for both research and educational purposes (design file 2).

### 2.2 Principle of ultrasonic sensing

**Fig. 4:**
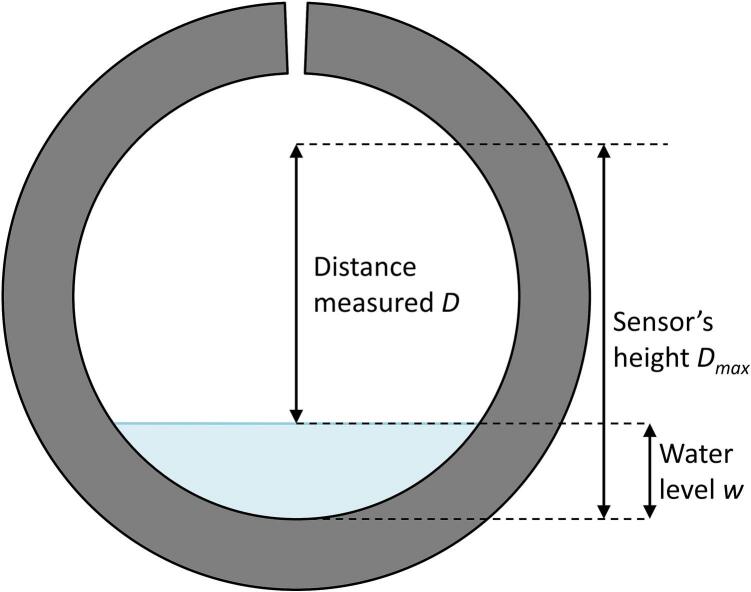
Principle of ultrasonic water level measurement in a pipe, with D_max_ the distance between the sensor and the bottom of the pipe, w as the water level and D the distance between the water surface and the sensor.

The low-cost ultrasonic sensor JSN-SR04T was selected from the available low-cost sensors on the market because it offers the best price *vs.* performance ratio and features waterproofing, an important characteristic for a sensor used in a wet environment. The distance measurement between the sensor and the water surface (denoted as *D* in Fig. 4) is performed when the TRIG pin receives a signal, triggering the emission of eight consecutive square pulse waves within 20 µs. The sensor then switches to “receiver mode” by setting the ECHO pin to a “HIGH” level until the sound wave returns. The time-of-flight *t* corresponds to the duration it takes for the wave to travel the distance *D* twice: from the sensor to the water surface and back (Eq. (1). The speed of sound *C* in air depends on the air temperature θ along the wave's trajectory and the relative humidity *RH,* as described in Eq. (2) from Panda et al. (2016) [4], which can be simplified to Eq. (3).

(1) $$D=C\times \frac{t}{2\times 10^{6}}$$

with *D* in m, *C* in m·s^−1^ and *t* in µs.

(2) $$C=\left(331.296+\theta \times 0.606\right)\times \left(1+\left(RH\times 9.604\times 10^{0.032\times \left(\theta -0.004\times \theta^{2}\right)-6}\right)\right)$$

(3) $$C_{\mathrm{simplified}}=331.4+\theta \times 0.6+0.124\times RH$$

with θ the temperature in °C, *RH* the relative humidity in %, and *C_simplified_* the sound velocity in m·s^−1^.

Compared to air temperature, air relative humidity has a much lower influence on the speed of sound (within the expected accuracy). Moreover, low-cost humidity and temperature sensors (*e.g.*, DHT11 or DHT22) have an accuracy of ± 5 % *RH* and are prone to deterioration when exposed to a wet environment [20,21]. Therefore, we decided to calculate sound velocity only by using the air temperature measurements made with a waterproof temperature sensor (DS18B20), and not to monitor air relative humidity, thus enhancing the robustness of *LevelWAN*. By transforming Eq. (2) into Eq. (3), we obtain a linear approximation in which the maximum uncertainty in sound velocity is 12.4 m/s, based on the difference between the cases when the air is either dry (*RH* = 0 %) or saturated with water (*RH* = 100 %). This results in an impact of $\Delta_{D\left(RH\right)}=6.2\times 10^{-6}\times t$ (with *t* in µs and Δ*_D_*_(_*_RH_*_)_ in m) on the calculation of distance *D*. According to Cherqui et al. [17], the maximum error without *RH* correction is 23 mm for a 100 % difference in *RH*. Since the device is designed for use in a wet environment, we can assume that the relative humidity remains high by arbitrarily using an 80 % *RH* value for the calculations, which helps reduce the error associated with *RH* while avoiding the need for an additional sensor.

Finally, the water level (denoted as *w* in Fig. 4a) is calculated as the difference between the maximum distance (*D_max_*) and the distance (*D*) measured by the sensor at a given time. *D_max_* must be measured on-site after the sensor is installed and serves as a reference for calculating variations in water level. For small rivers or pipes with depths less than 2 m, *D_max_* is determined by the riverbed conditions. Therefore, the height should be checked regularly (*e.g.*, after each flood) due to potential sedimentation or erosion of the cross-section.

### 2.3 Arduino script description

**Fig. 5:**
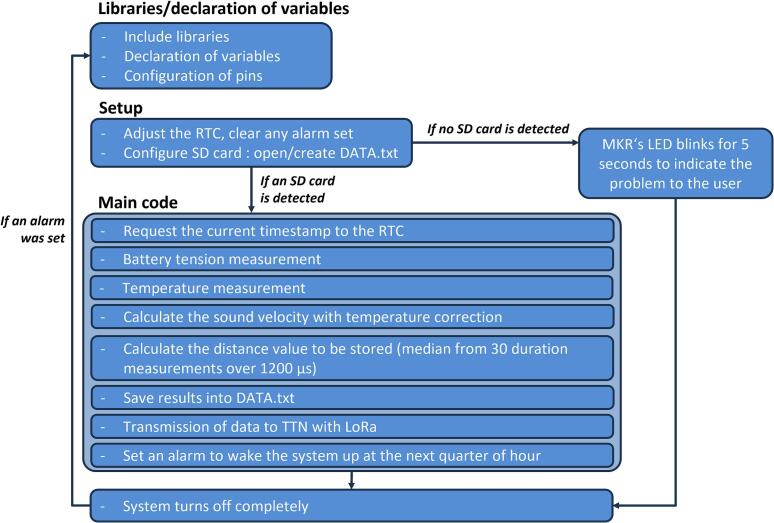
Architecture of the LevelWAN script executed by the device software, with the different sections.

The file “Code_LevelWAN.ino” (design file 2 available in the repository) is an Arduino script written in C++. It comprises three main sections (Fig. 5). The first one, “Libraries and Variable Declarations” (Fig. 5), includes calls to the libraries associated with the electronic components used:

• MKRWAN.h, for sending data via LoRaWAN;
• ds3231.h and RTClib.h, for the RTC module;
• Wire.h, for devices communicating data using the I2C protocol;
• OneWire.h and DallasTemperature.h, for the temperature sensor;
• SD.h and SPI.h, for saving data to the datalogger;
• RunningMedian.h, for extracting the median value of a series to reduce uncertainty in the measured distance.

In this section, the user must define the recording time step (in minutes), which determines the interval between two consecutive measurements, by setting the variable “sleep_period” (Table 3). A default value is set to 15 min to comply with the recommendations of the LoRaWAN protocol.

**Fig. 6:**
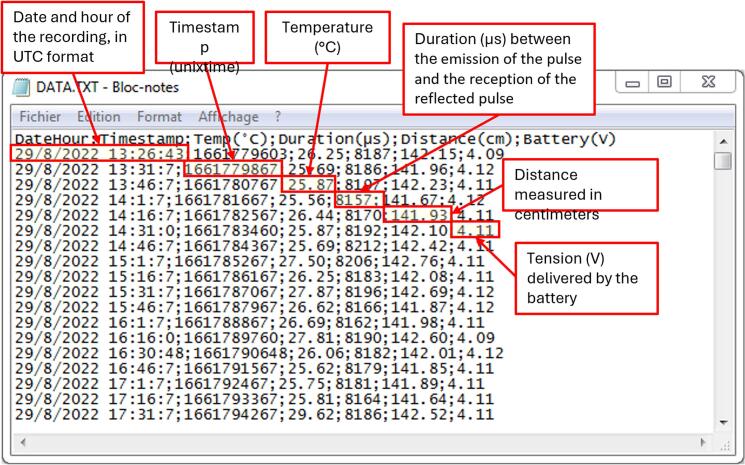
Example of output text file locally saved on SD card.

In the “Setup” section (Fig. 5), the system is initialized by assigning names and modes to each pin used by the various modules. A file named DATA.txt (Fig. 6) is created on the SD card with the following headers: “DateHour; Timestamp; Temp(°C); Duration(µs); Distance(cm); Battery(V)”. As a reference, 10 months of data recorded at 15-minute intervals require approximately 1.5 MB of storage. The program is designed to prevent the system from starting without an SD card due to potential unreliability in online data transmission. If the SD card is present and the DATA.txt file already exists, new measurements are appended to the end of the file at each time step. If the SD card is missing or improperly inserted, the device indicates the issue by blinking a yellow LED (Fig. 5) before shutting down automatically to conserve battery power.

The “Main Code” section (Fig. 5) details the *loop()* function executed by the microcontroller to measure all required quantities: battery voltage, air temperature, and the distance *D* of the ultrasonic sensor based on the time-of-flight of the sound wave (Table 3). These values, along with the timestamp, are first stored on the SD card and then transmitted to TTN. To minimize uncertainty, a series of 30 time-of-flight measurements is performed using a “*for*” loop. At each iteration, an “*if*” condition excludes values below 1200 µs, creating a list of 30 valid measurements from which the median is calculated. A 1200 µs threshold is applied because ultrasonic sensors are generally unreliable at very short distances due to a built-in “dead zone” immediately after signal emission [9], and the reliable measurement range is defined between 0.2 and 2 m [17]. At close range, the echo may return too quickly, overlapping with the outgoing pulse or being missed entirely. Additionally, the sound beam is wide at short distances, reducing spatial accuracy and increasing measurement noise. Strong or poorly reflected signals can also saturate or confuse the receiver, leading to inconsistent readings.

Before the loop concludes, the next alarm is set to wake the system at regular intervals, *e.g.*, every 15 min (XX:00, XX:15, XX:30, XX:45). The *loop()* function executes only once per cycle, as the system is designed to power down at the end of each sequence to save energy.

## 3 Design files summary

• Design file 1 is a compressed folder containing all the files needed to reproduce the *LevelWAN* PCB.
• Design file 2 is the program for the MKR WAN 1310, mentioned in section 2 and shown on Fig. 8 (Arduino IDE is needed to upload the file).
• Design file 3 is a video tutorial providing the visual instructions detailed in section 5. The audio is available in French, English and Italian.
• Design file 4 is the JavaScript code needed to transfer data from TTN to Google Sheet.

## 4 Bill of materials summary

This system is designed around every specific element mentioned in Table 2 with their indicative price shipping costs not included (may vary depending on the region). All connections are designed to be simple enough for non-professionals, minimizing the need for free wires and soldering. In line with the low-cost and low-tech approach, it is recommended, where feasible, to reuse components from previous devices, such as wires, resistors, or enclosures similar to the PVC used in this project. For example, a 1000 mL Nalgene® container would be sufficient to house all components along with a small packet of silica beads to absorb humidity. This Nalgene® container costs €10.07 in Europe (ASMC, article code: 80812) or $6.29 in North America (available at Nalgene). Unlike PVC-based enclosures, this solution does not require PVC cement. However alternatives can still be purchased depending on users’ will, but we cannot guarantee full compatibility either for the sensing part or the datalogging part. As for other controllers that could also be used, every other version of Arduino’s MKR is expected to work perfectly fine with the LevelWAN PCB, but the user would have to adapt the code depending on the chosen network instead of LoRaWAN (e.g., SigFox, NB-IoT) to properly send data online. In fact, most of these controllers are fully compatible with a similar datalogging system developed by Perret et al. [22].

**Table 2:** Bill of materials (indicative prices at 03.03.2025 in France, shipping costs may vary depending on the country).

**Designator**	**Component**	**Number**	**Cost per unit -Euros**	**Total Cost -Euros**	**Source of materials**	**Material type**
**Ultrasonic sensor**	JSN-SR04T-V3.0 sensor	1	18,20 €	18,20 €	Gotronic Article code: 35008 https://www.gotronic.fr/art-capteur-etanche-a-ultrasons-sen0208-25729.htm	Other
**Temperature sensor**	DS18B20 sensor	1	7,95 €	7,95 €	Gotronic, Article code: 31695 https://www.gotronic.fr/art-sonde-etanche-ds18b20-19339.htm	Other
**Microcontroller**	Arduino MKR WAN 1310 controller	1	49,50 €	49,50 €	RS, Article code: 202-4162 https://fr.rs-online.com/web/p/outils-de-developpement-pour-microcontroleurs/2024162?gb = a	Other
**Antenna**	LoRa telecommunication antenna	1	5,15 €	5,15 €	RS, Article code: 169-7591 https://fr.rs-online.com/web/p/shields-pour-arduino/1697591	Other
**Shield SD**	Module SD Arduino MKR Mem Shield	1	24,23 €	24,23 €	RS, Article code: 176-3644 https://fr.rs-online.com/web/p/shields-pour-arduino/1763644?gb = s	Other
**MicroSD card**	MicroSD card 16 GB	1	9,54 €	9,54 €	RS, Article code: 180-5793 https://fr.rs-online.com/web/p/cartes-sd/1805793	Other
**Battery**	Accu LiPo 3.7 Vcc 4000 mAh	1	23,50 €	23,50 €	Gotronic, Article code: 09948 https://www.gotronic.fr/art-accu-lipo-3–7-vcc-4000-mah-l805080-31843.htm	Other
**Battery module**	Lipo rider pro card	1	15,60 €	15,60 €	Gotronic, Article code: 31355 https://www.gotronic.fr/art-carte-lipo-rider-pro-106990008-19050.htm	Other
**Wire**	Wire to be soldered to the LiPo Rider Pro	∼5 cm	0,85 € /meter	0,05 €	RS, Article code: 201-2709 https://fr.rs-online.com/web/p/fils-de-cablage/2012709	Other
**Solar panel**	Solar Cell SOL3W	1	19,00 €	19,00 €	Gotronic, Article code: 27134 https://www.gotronic.fr/art-cellule-solaire-sol3w-18996.htm	Other
**Waterproof wires**	Waterproof extension for the red and black wires of the solar cell	1	10,86 €	10,86 €	RS, Article code: 253-7022 https://fr.rs-online.com/web/p/cables-d-alimentation/2537022	Other
**USB cables**	USB A − micro-USB A M/M cable	2	1,95 €	3,90 €	Gotronic, Article code: 48314 https://www.gotronic.fr/art-cordon-50-cm-rs105-33657.htm	Other
**LevelWAN PCB**	Printed Circuit Board with components **(Design file 1)**	1	∼65 € / 5 pieces	∼13 €	JLC PCB: https://jlcpcb.com	Other
**Flat battery**	Battery 3 V CR2032	1	1,14 €	1,14 €	RS, Article code: 186-3376 https://fr.rs-online.com/web/p/piles-boutons/1863376?gb = s	Other
**PVC pipe**	PVC Pipe (4 cm diameter) to stick the solar panel to	1	3,59 €	3,59 €	Castorama, ref: 3396040110401 https://www.castorama.fr/tuyau-en-pvc-diametre-40-mm-x-1-m-fitt/3396040110401_CAFR.prd	Other
**PVC sleeve**	PVC sleeve FF: 10 cm diameter	1	1,70 €	1,70 €	Castorama, ref: 3396047440105 https://www.castorama.fr/manchon-a-butee-pvc-evacuation-100-fitt/3396047440105_CAFR.prd	Other
**Watertight PVC inspection cover**	PVC inspection cover: 10 cm diameter	2	3,70 €	7,40 €	Castorama, ref: 3396047450104 https://www.castorama.fr/tampon-de-visite-male-diam-100-mm-fitt/3396047450104_CAFR.prd	Other
**PVC cement**	Glue for PVC pipe 125 g	1	5,34 €	5,34 €	RS, Article code: 379-8817 https://fr.rs-online.com/web/p/colles-de-tubes-plastiques/3798817?gb = s	Other
**Cable glands**	Pack of 10 waterproof cable glands for 5 to 10 mm cable	3 out of 10	17,59 €	5,28 €	RS, Article code: 822-9653 https://fr.rs-online.com/web/p/presse-etoupes/8229653	Other
**TOTAL**	/	/	/	208,79 €	/	/

**Table 3:** Main variables of the Arduino program (Design File 2).

Variable name	Type	Description
appEui	char	AppEUI/joinEUI to define on TTN
appKey	char	“AppKey” to generate on TTN when registering the microcontroller to a LoRaWAN application in the network.
SWITCH_ON_OFF	int	Indicates the MKR pin used to keep the system ON and turn OFF by either setting the pinmode to HIGH or LOW respectively
vBat	float	Battery tension value
tempValue	float	Temperature measurement made with DS18B20
samples	RunningMedian	An array to store the 30 duration measurements and get the median value out of them
duration	float	The time-of-flight measurement made by JSN-SR04T
distanceValue	float	The actual distance calculated from duration and tempValue
RHValue	float	Relative humidity value set to 80 % by default

## 5 Build instructions

**Fig. 7:**
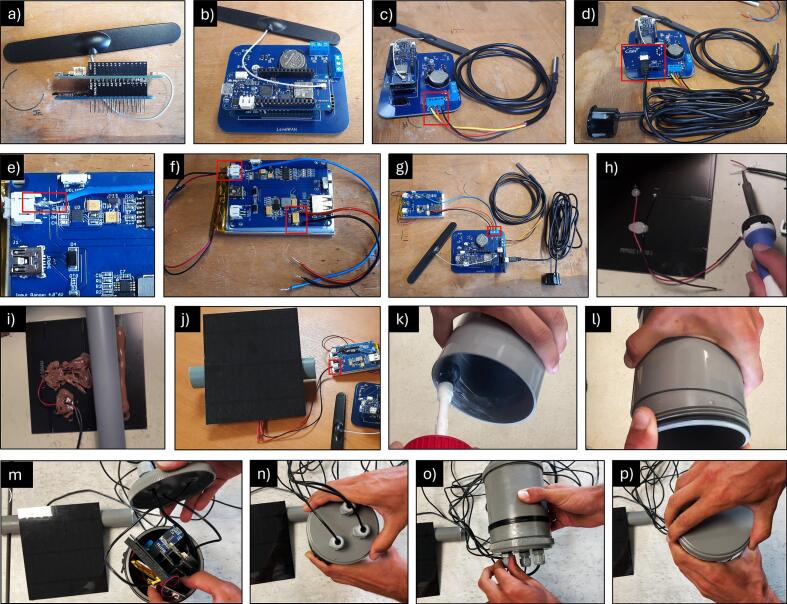
Instructions to assemble the complete system in 16 steps. They are also available as a video tutorial in French, English and Italian (design file 3).

The following 16 steps detail the connections between the components of LevelWAN (Fig. 1) and correspond to the visual instructions in Fig. 7:

**Fig. 8:**
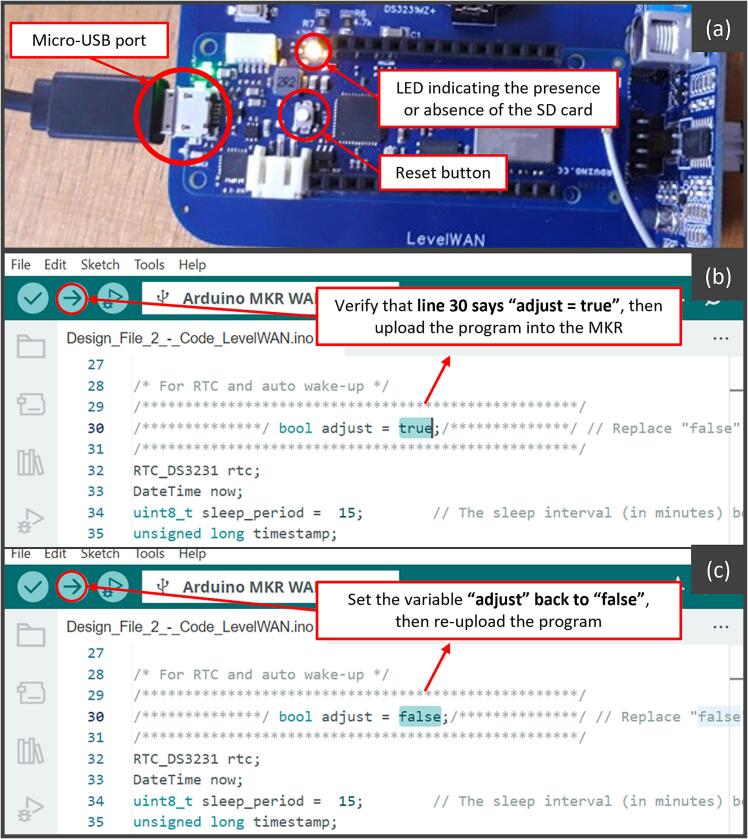
Uploading the program to the MKR WAN 1310 (a) and adjusting the time on the RTC module.

**Step 1** (Fig. 7a): plug the MKR WAN 1310 onto the MKR SD Mem Shield (or vice versa) and attach the LoRa antenna to the MKR WAN 1310 microcontroller;

**Step 2** (Fig. 7b): plug the MKR devices to the PCB;

**Step 3** (Fig. 7c): connect the three wires of the DS18B20 temperature sensor to the PCB slots labeled “TEMPERATURE” using a screwdriver, ensuring wire connections match Table 4;

**Table 4:** Connections to the MKR.

Sensor	Inputs and outputs	MKR pin
JSN-SR04T	GND(−)	GND(−)
	5 V	5 V
	ECHO	0
	TRIG	1
DS18B20	GND(−)	GND(−)
	VCC(+)	VCC(+)
	S	2
LiPo Rider Pro	GND(−)	GND(−)
	VCC(+)	VCC(+)
	vBat	A1

**Step 4** (Fig. 7d): connect the ultrasonic sensor module to the header row labeled “SR04T” on the PCB, ensuring the GND (−) pin aligns with the board’s markings;

**Step 5** (Fig. 7e): the battery regulator requires a wire to be soldered to the VCC (+) pin of the JST connector receiving the LiPo battery. This wire is used to monitor the battery voltage (Fig. 1);

**Step 6** (Fig. 7f): two extra wires need to be soldered on the VCC (+) and GND (−) of the C13 condenser to get the output 5 V tension (a voltmeter is recommended for identification). Next, keep the LiPo Rider Pro switched OFF and connect the battery to the upper JST port. All wire ends need to be tinned before soldering or inserting them inside the screw slots of the PCB;

**Step 7** (Fig. 7g): by connecting the LiPo Rider Pro onto the PCB, the assembled LevelWAN system is ready for testing. Verify the ultrasonic sensor detection, temperature measurement, and auto wake-up functionality before field deployment;

**Step 8** (Fig. 7h): extend the solar panel wires by desoldering the original short wires of the SOL3W and soldering them to the two wires of a waterproof cable for better placement flexibility;

**Step 9** (Fig. 7i): glue a PVC pipe (20 cm long; 3 cm diameter) to the back of the solar panel to facilitate optimal on-site positioning;

**Step 10** (Fig. 7j): connect the solar panel to the battery manager via the “Solar Adapter 5 V” JST port;

**Step 11** (Fig. 7k): assemble the PVC sleeve with its two covers to create a weatherproof box. Apply PVC cement to both ends of the sleeve to secure the inspection covers;

**Step 12** (Fig. 7l): insert the inspection covers on both ends of the sleeve;

**Step 13** (Fig. 7m): place all previously assembled components inside the PVC sleeve;

**Step 14** (Fig. 7n): drill three or four holes in one of the inspection covers and install the corresponding cable glands (Fig. 3). Glue them to the box. These glands will allow the waterproof cables to exit the box: (1) the solar panel cable, (2) the ultrasonic sensor cable, (3) the temperature sensor cable, and optionally, (4) an extension for the LoRa antenna;

**Step 15** (Fig. 7o): insert the cables through the glands and securely tighten them;

**Step 16** (Fig. 7p): finally, tightly seal the box to ensure it is weatherproof, protecting the system from rain, moisture and insects.

## 6 Operation instructions

### 6.1 Before installation

The script must be uploaded to the microcontroller, and the RTC (Real-Time Clock) must be set to the current time. To do this, connect the MKR WAN 1310 to a computer using a micro-USB cable (Fig. 8a). Open the file “Code_LevelWAN.ino” in the Arduino Integrated Development Environment (IDE).

First, ensure all required libraries are installed, and set the Boolean variable “adjust” to “true” line 30 (Fig. 8b). Then, upload the program to the microcontroller using the appropriate command (Fig. 8b). During this process, the RTC synchronizes its clock to the computer’s system’s time. To ensure accuracy, it is recommended that the computer’s clock is set to UTC. After successfully uploading the script, and without disconnecting the micro-USB cable, change the “adjust” variable to “false” as shown in Fig. 8c and re-upload the modified script. This two-step method prevents the system from resetting its clock to the same time every time it powers on. As the RTC may experience slight time drift over long periods (up to 1 min per year, according to the manufacturer [19]), a GPS module can be integrated to periodically resynchronize the RTC, for example, once per month. The addition of such module is not recommended since the power consumption of an Arduino-compatible GPS module would be relatively high (>30  mA for several minutes). Typically it would require either the use of its own dedicated flat battery or controlled power management by the MKR board once per day.

### 6.2 Data management with TTN (The Things Network)

**Fig. 9:**
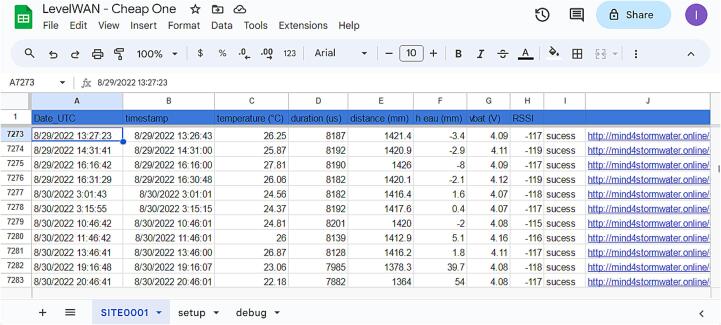
Google sheet file with LoRa sent data (script available in design file 4).

The LoRa antenna enables the transmission of data to The Things Network (TTN), an open-source platform that manages LoRaWAN communication. Data received by TTN is updated at each time step in an online file (Fig. 9). The recorded values can be visualized using third-party platforms such as Node-RED, Grafana, or InfluxDB, or, as in this study, on the Opendataeau website (https://opendataeau.org/).

One must pay attention that while the LoRaWAN network offers strong built-in security features, such as end-to-end encryption and device authentication, it is not invulnerable and must be complemented with proper implementation and monitoring practices. For instance, users should ensure robust key management and regular review of network access permissions as part of these practices. The open-source sharing of time series data, especially in citizen science projects, also requires careful attention to privacy concerns. Such water level data may contain sensitive information, such as precise locations or patterns of activity. In Europe, compliance with the General Data Protection Regulation (GDPR) is essential, along with proper anonymization and participant awareness before publication.

The file contains all the quantities measured and transmitted by the device, organized into columns B, C, D, E, and G. These correspond to the same data described in Fig. 6, with the addition of five new columns (A, F, H–K) that facilitate remote monitoring:

• **Column A** includes a timestamp indicating when the data was received on the server, allowing the calculation of the delay between the local save (on SD card) and the TTN reception time.
• **Column F** provides the calculated water level (in millimeters), derived from the measured distance *D* using Eq. (1).
• **Column H** records the Received Signal Strength Indication (RSSI), which represents the strength of the LoRaWAN signal at the gateway. A strong signal typically measures around –80 dBm, while a weak signal is closer to –120 dBm [23].
• **Columns I and J** are used to transmit data to Opendataeau.org and confirm successful communication. Column I indicates whether the data transmission was successful (“success”) or failed. In cases of failure (*e.g.*, due to high server traffic), the link remains active for a retry. Column J generates a unique HTML link for each measurement, which is automatically opened to send the data to the platform. The URL includes: i) an HTTP address to connect to the platform, ii) a secret key to verify the legitimacy of the transmission, iii) a “box” parameter identifying the measurement location, iv) several “code” and “val” pairs representing parameters and their corresponding values, v) and a final parameter indicating the measurement timestamp (in Unix time).

### 6.3 Field instructions

The system must be installed in a secure location near the measurement point to ensure that the wires are not overstretched. The installation site should also facilitate effective radio communication, preferably elevated and not isolated within concrete structures or buildings.

The JSN-SR04T sensor is equipped with a 2.4 m cable, allowing flexibility in positioning. The distance between the sensor’s emitter/receiver and the target surface should be adjusted before securing the sensor. The sensor must be mounted perpendicular to the water’s free surface using a metallic bracket or a plastic strap attached to a pole or tree. It should face downward and be positioned away from any surfaces that might reflect acoustic signals, as such reflections could interfere with the primary measurement signal. The temperature probe should be secured with the same strap, placed as close as possible to the ultrasonic emitter/receiver to ensure accurate measurements. The solar panel should be inclined using the attached PVC pipe to optimize exposure to sunlight; the ideal angle depends on the installation site's latitude.

Once the setup is complete, the user must measure or calculate the maximum distance (*Dmax*) between the ultrasonic emitter/receiver and the bottom of the canal when it is dry. This value is crucial for data analysis and is required for the Google Sheet script (design file 4). Alternatively, if a water gauge is installed near the measurement point, its readings can be used as a reference.

It is also essential to verify the presence of a nearby LoRaWAN gateway to ensure data transmission to The Things Network (TTN). If no gateway is available, data will only be stored locally on the SD card. For this reason, the system should be installed in an easily accessible location, as the SD card will need to be regularly accessed for data retrieval and archiving. Before powering on the system, open the PVC enclosure and verify the following:

• The program has been successfully uploaded (Fig. 8a);
• The RTC time is correctly set (Fig. 8b; Fig. 8c);
• The formatted SD card is properly inserted into the MKR Shield. If not, the MKR indicator light will blink for 5 s.

Once all checks are complete, the user should switch the Lipo Rider’s switch (SW1) to the “ON” position. If the collection interval is set to 15 min, data collection will begin at the next quarter-hour mark. Although not mandatory, the user can manually trigger a measurement by holding down the “SW” button on the PCB for 30 s. It is recommended to perform a test measurement to ensure the device is functioning properly during maintenance or data archiving. To remove the SD card, the user must open the inspection cover without cable glands and turn off the Lipo Rider beforehand.

No safety hazards related to the operation of the hardware were identified. However, potential risks may of course vary depending on the installation location.

## 7 Validation and characterization

### 7.1 Field description and reference sensors

The core component of the system is the JSN-SR04T sensor, previously calibrated under controlled conditions with an expanded uncertainty of 95 %, resulting in an estimated error of 7 mm [17]. However, this sensor had not been tested in field conditions or over extended periods (i.e., beyond several months). The first *LevelWAN* station was assembled and deployed in late 2020 in order to evaluate the system's reliability under these conditions. Since its installation, the system has operated autonomously at 15-minute intervals, with occasional adjustments to its placement and software updates related to water level calculations.

**Fig. 10:**
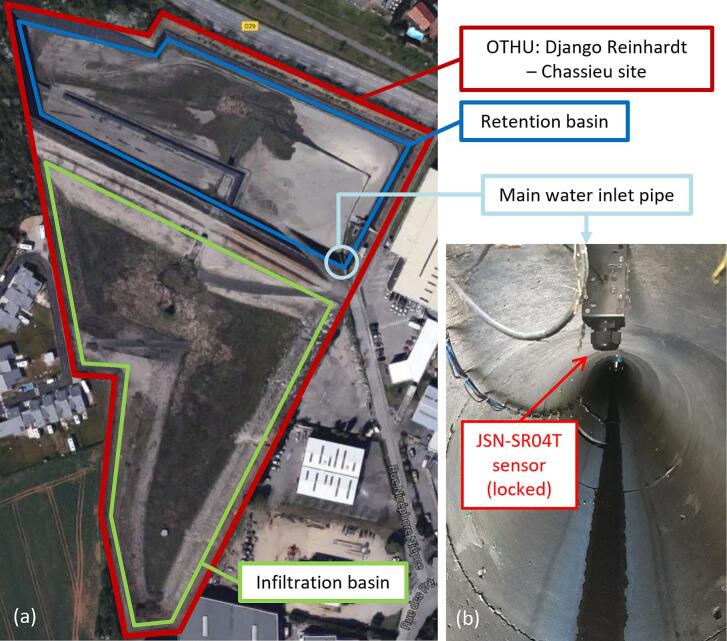
Location of the sensor at the OTHU Django Reinhardt study site (a), inside the inlet sewer pipe (diameter = 1.6 m) (b).

LevelWAN was installed at a study site managed by the OTHU (https://www.othu.org – Field Observatory for Urban Water Management; site name: Django Reinhardt). This site includes stormwater management infrastructure designed for an industrial catchment in Chassieu, France, comprising a retention basin and an infiltration basin (Fig. 10a), with many other sensors and a LoRaWAN gateway (model: The Things Gateway [24]) already installed ∼ 50 m away from our station. The monitoring system was placed inside the main inlet sewer pipe (1.6 m in diameter) of the retention basin (Fig. 10b). The JSN-SR04T emitter was positioned at a height *Dmax* = 1.43  m above the pipe's bottom. A VEGAPULS WL 61 radar sensor (Table 5; 95 % expanded uncertainty of ± 2.0 mm [25]) monitored the water level in the sewer pipe at a 2-minute time-step interval from August 29th, 2022, to August 31st, 2023. *LevelWAN* was installed 5 m downstream of this reference sensor to evaluate its performance during this period.

### 7.2 Sensor comparison and assessment

**Fig. 11:**
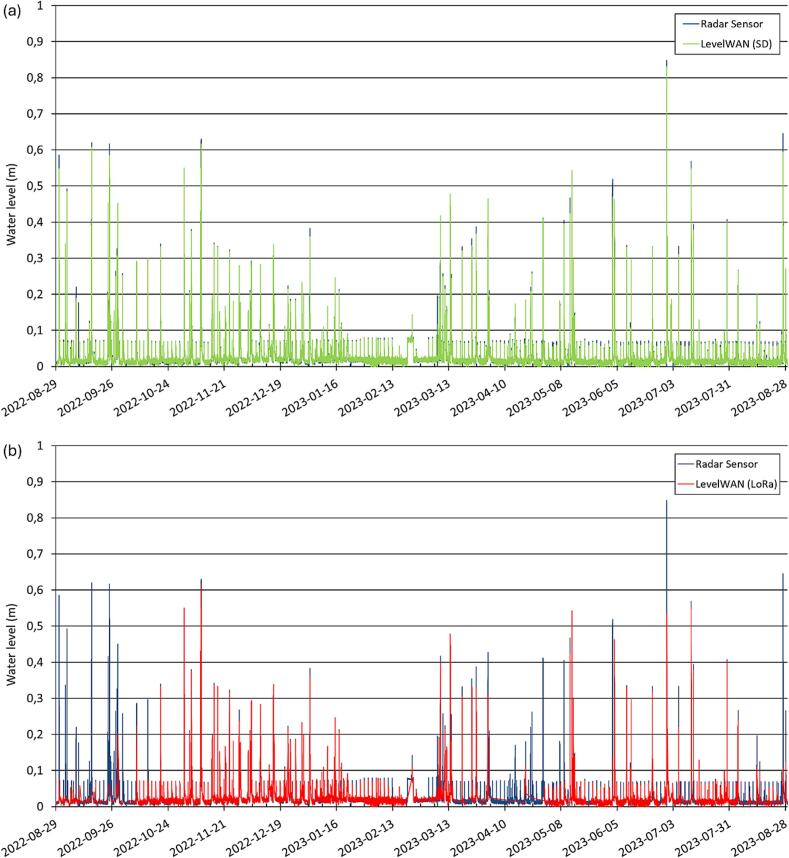
Water level monitoring in the sewer pipe from August 29th, 2022 to August 31st, 2023. Comparison of raw-data from the radar sensor (in blue for both figures) and LevelWAN system: i) data stored in the SD card (green; Fig. 11a) and ii) the data measured by the LevelWAN transmitted by the LoRaWAN network (red; Fig. 11b). (For interpretation of the references to colour in this figure legend, the reader is referred to the web version of this article.)

The raw data transmitted by *LevelWAN* via LoRaWAN reveal significant data losses (Fig. 11b). Of the 35,275 measurements recorded locally on the SD card, 27.479 were successfully transmitted online, representing approximately 20 % data loss. This loss is mainly due to a deactivation of OTHU’s LoRa gateway from April 2nd to April 29th in 2023, but also to a random connection problems throughout the field study with no explicit pattern identified. This highlights the critical importance of locally saving data on an SD card. While online data provide real-time access to system performance and facilitate quick visualization of recent events, comprehensive analyses require the complete dataset stored on the SD card. If needed, there could be a possibility to implement the creation of a temporary file on the SD card to store not transmitted data, and try again sending them again later. But for *LevelWAN*, online data availability is not meant to be the primary way of saving, plus sending too many messages under a short period of time goes against the LoRa protocol recommendations.

The performance of *LevelWAN* was first assessed by comparing its time series with those from the reference radar sensor (Fig. 11). As shown in Fig. 11a, the two time series exhibit an almost perfect overlap. However, notable discrepancies occur during two short-duration rainfall events (September 11th, 2022, and March 7th, 2023), lasting less than 15 min each. During these events, only the radar sensor, with its shorter 2-minute time-step, successfully captured the rapid flood dynamics, whereas *LevelWAN*, with its longer 15-minute time-step, failed to do so.

**Fig. 12:**
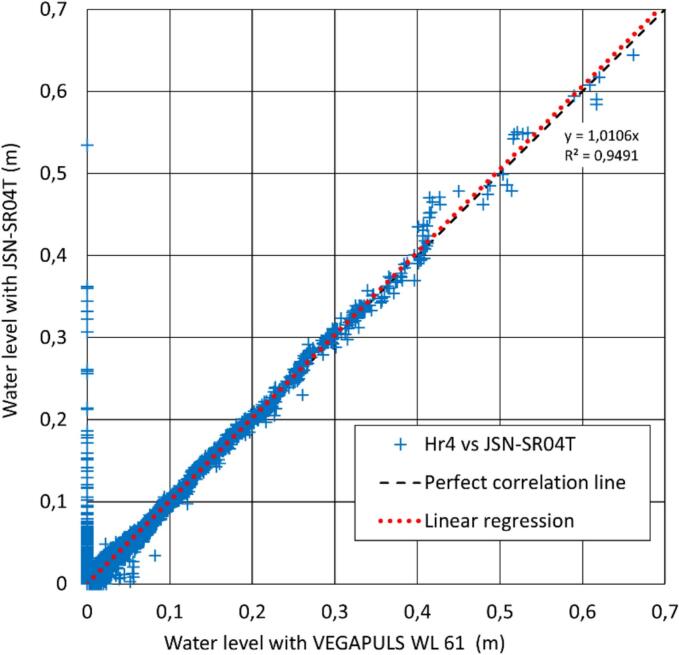
Comparison of water level measurements between LevelWAN and the radar sensors based on 35,066 paired data points.

Between August 29th, 2022, and August 31st, 2023, 35.066 data pairs from LevelWAN and the radar sensors were identified with matching timestamps, allowing for direct comparison. Of these, 95.9 % of *LevelWAN* measurements deviated by less than 1 cm from the radar sensor recordings (Fig. 12). The calculated root-mean-square error of the dataset is 9.9 mm and a linear regression (intersection set to zero) shows an equation of y = 1.0106x, with a correlation coefficient R^2^ = 0,9491. This performance demonstrates that *LevelWAN* is suitable for operational use in most water-related applications, such as monitoring rivers, lakes, basins, and sewer pipes. However, perfect correlation between the two sensors is inherently unattainable for at least two reasons: (i) the measurement time steps differ, and (ii) the sensors are positioned 5 m apart, which inevitably introduces some discrepancies. A small subset of measurements is less satisfactory, with water level differences ranging from 0.01 m to 0.38 m (most of them are less than 10 cm). Additionally, for water level measurements over 0.4 m, Fig. 12 shows a waving pattern very likely due to local conditions since OTHU technicians also witnessed the same motif from one VEGAPULS to the other. Further investigation is required to fully understand these discrepancies and identify their causes. One minor possible explanation could be the omission of relative humidity (*RH*) correction for LevelWAN, or the oversimplified Eq. (3)used instead of Eq. (2).

### 7.3 RTC auto-wake up precision

**Fig. 13:**
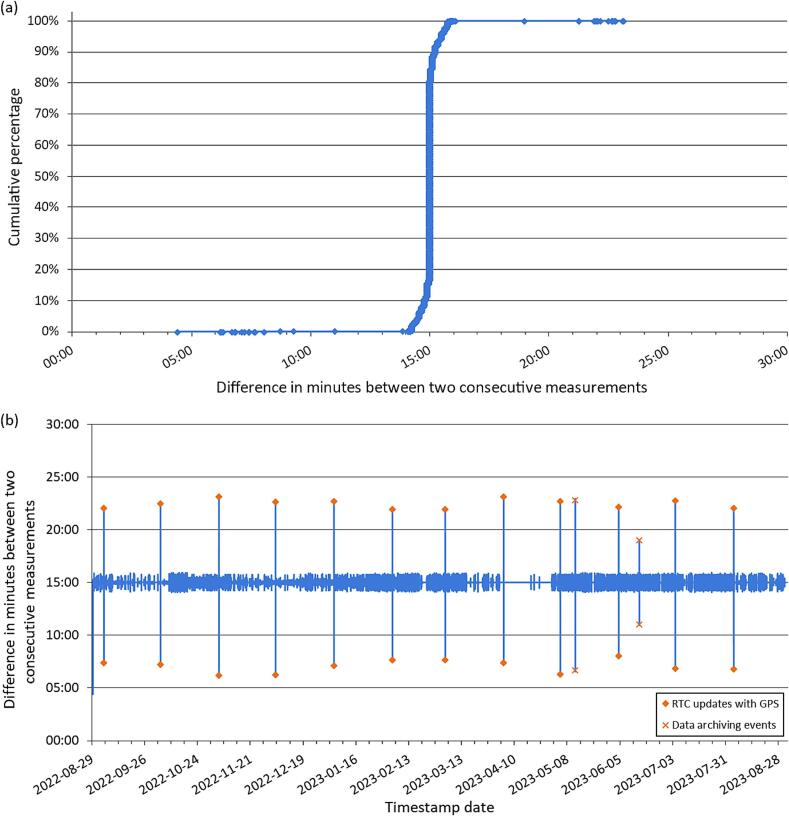
Time intervals between successive measurements from August 29, 2022, to August 31, 2023: (a) cumulative percentage of measurements per timespan; and (b) evolution of the difference between consecutive measurement timestamps.

The time intervals between successive *LevelWAN* measurements saved on the SD card were analyzed to identify potential long-term temporal drifts in the system (Fig. 13). Configured for a 15-minute interval, 99 % of the recorded intervals fall within the range of 14 to 16 min (Fig. 13a). While not perfectly consistent, this performance is deemed acceptable for most operational uses and water-related applications.

The time interval cannot be strictly constant due to the time required to establish a connection with the LoRa gateway, which occurs during the setup phase of the code prior to saving the current timestamp (Fig. 5). Additionally, two measurements (on May 12th, 2023, and June 15th, 2023) exhibited different intervals due to field visits involving SD card downloads, which delayed the measurements by activating the “SW” switch on the PCB (Fig. 2, Fig. 4).

### 7.4 LoRaWAN “near real-time” reliability

**Fig. 14:**
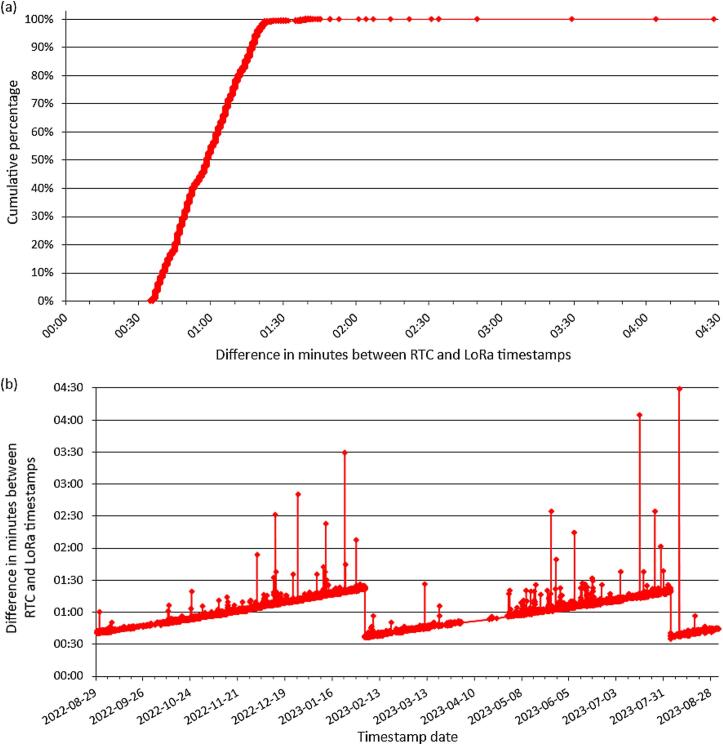
Delay between the collection of the data on SD card (i.e., RTC timestamp) and its reception online with LoRa during the assessment period (i.e., LoRa timestamp): (a) cumulative percentage of measurements; (b) evolution of the difference sbetween the measurement timestamp and the gateway timestamp.

It is expected to have a delay between the measurement time and the time the message is received by the LoRaWAN gateway, mainly because of the distance between the device and the gateway. We analyzed here the time interval between the timestamps of the SD card and the timestamp for the data received online by using the LoRaWAN (Fig. 14). This time difference varies, in relation to the quality of the communication (RSSI = Received Signal Strength Indication). Very poor communication (RSSI close to −120 dB) could take up to three minutes to convoy the message (Fig. 14a), while less than 30 s are needed with a good quality signal.

Moreover, Fig. 14b shows the evolution of the difference over time. Although the difference varies over time, there is a gradual increase that corresponds to the drift of the internal clock (RTC). The RTC has been adjusted only two times (2023-02-04 and 2023-08-04). The average drift of the RTC is around 7 to 8 s per month, corresponding to the drift rate of about 1 min per year announced by the datasheet [19].

### 7.5 Battery level and power consumption

**Fig. 15:**
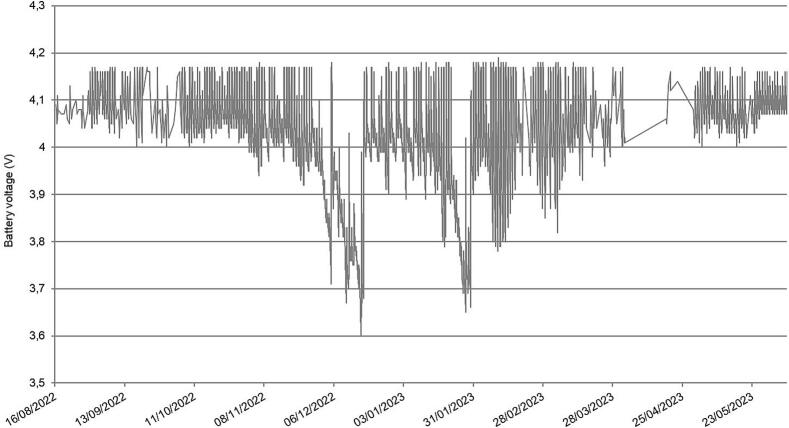
Battery voltage for LevelWAN during the field test period.

Since the system depends on solar energy to recharge the LiPo battery, winter represents of course the most critical period. Fig. 15 illustrates the capability of *LevelWAN* to operate continuously and autonomously over an entire year, including during winter, with the battery voltage consistently remaining above 3.6 V.

The performance of the auto wake-up mode was evaluated in the laboratory using only a LiPo Rider without a solar panel. This single test aimed to estimate the battery life and the total number of measurements the system could achieve in the event of a solar panel failure in the field, starting with a fully charged battery. The system operated for 42 days with a total of about 4,000 recordings.

## 8 Conclusion

An autonomous, open-source, and cost-effective IoT-based water level monitoring system has been developed, integrating an ultrasonic sensor to provide near real-time online data at an approximate cost of €200. This represents a significant cost reduction compared to traditional systems, which typically range from €2,000 for basic models to over €4,000 for high-end alternatives offering similar functionalities.

LevelWAN has demonstrated performance comparable to conventional systems across several key parameters: (i) robustness, with more than three years of continuous in situ operation without failure; (ii) a sampling frequency of 15 min, sufficient for most hydrological applications, with the option to increase resolution to 5-minute intervals; (iii) efficient power management, where the integrated solar panel reliably recharges the battery as needed; (iv) validation through laboratory experiments and a year-long field assessment. The system required minimal maintenance, with field visits necessary only once every four months for data backup from the SD card and resetting of the Real-Time Clock (RTC).

Designed for ease of assembly, *LevelWAN* is well-suited for both research and educational applications. This was confirmed during metrology courses at the University, where Master’s students with limited experience in electronics, metrology, and soldering successfully built and deployed the system. This study thus highlights *LevelWAN* as a reliable, low-cost alternative for water level monitoring, supporting citizen science, environmental research, and educational outreach.

Specifications table

**Table:** 

Hardware name	*LevelWAN*
Subject area	• Engineering and materials science • Environmental, planetary and agricultural sciences • Educational tools and open-source alternatives to existing infrastructure
Hardware type	• Field measurements and sensors • Mechanical engineering and materials science
Closest commercial analog	Some analog sensors (without datalogger): • Delta Mobrey 003S0/M03 • Delta Mobrey MSP900GH-A • Siemens Milltronics MiniRanger-plus • Nivus GmbH i-series • Hach Flo-Dar FL900, FL1500 • Paratronic US10 • *Milesight EM400-MUD-868 M PN: B045-2 (low-cost IoT system)*
Open-source license	GNU General Public License (GPL) 3.0
Cost of hardware	∼200 €
Source file repository	https://osf.io/4nw8s

## CRediT authorship contribution statement

**Ilane Cherif:** Writing – review & editing, Data curation. **Frédéric Cherqui:** Writing – review & editing, Software, Resources, Investigation. **Franck Perret:** Software, Resources, Methodology. **Bastien Bourjaillat:** Writing – original draft, Validation, Software, Resources, Methodology. **Lionel Lord:** Validation, Software, Resources, Methodology. **Jean-Luc Bertrand-Krajewski:** Writing – review & editing, Software, Formal analysis. **Nicolas Walcker:** Validation, Data curation. **Maria Gisi:** Writing – review & editing. **Laëtitia Bacot:** Project administration, Funding acquisition, Writing – review & editing. **Oldrich Navratil:** Writing – review & editing, Supervision, Software, Project administration, Methodology, Funding acquisition, Conceptualization.

## Declaration of competing interest

The authors declare that they have no known competing financial interests or personal relationships that could have appeared to influence the work reported in this paper.

## Figures and Tables

**Table 5:** Radar sensor VEGAPULS WL 61 specifications ([25]).

	**VEGAPULS WL 61**
**Applications**	Water treatment, pumping stations, rainwater harvesting basins, flow measurement in open channels and level monitoring
**Max. measuring range**	15 m (49.21 ft)
**Antenna/Material**	Plastic cone antenna
**Process/material connection**	Threading G1½ /PBT or mounting bracket /316L
**Process temperature**	−40 … +80 °C −40 … +176 °F)
**Process pressure**	−1 … +2 bar/-100 … +200 kPa (−14.5 … +29.0 psi)
**Measurement deviation**	≤2 mm
**Frequency range**	K band
**Signal output**	• 4 … 20 mA/HART – two wires • Profibus PA • Foundation Fieldbus
**Display/Settings**	• PACTware • VEGADIS 62
**Approvals**	• ATEX • IEC

## Data Availability

Data will be made available on request.
